# Progress in Surgery

**Published:** 1895-08-17

**Authors:** 


### Progress in Surgery.

### ABDOMINAL SURGERY.

**The Parietal Incision.** — Greig Smith[1] formulates the following rules for making an abdominal incision: 1. The line of parietal incision should be made parallel with the direction of the most important muscular fibres. 2. Separate where possible, and do not divide aponeurotic fibres; where division is necessary, let it be in a direction which will permit of the leaving intact one or other of the muscular layers behind the division, or in front of it, or parallel to it. If no one of these practices is possible, then a flap entrance should be made, that is to say, the weak points do not all overlie; they are made at different levels in the parietes when union is made. 3. Keep away from the bony margins, and avoid the thickest and most mobile parts of the parietes. 4. Let the incision be as short as is consistent with efficiency. A long incision with separation of muscular and aponeurotic fibres is better than a short one with division of fibres. The leading principles for our guidance in the closing of a parietal incision are: 1. The apposition of raw surfaces should be as broad as possible. 2. Each divided structure should be placed and kept opposite its fellow. 3. The sutures should not be removed early. If the parts are kept together firmly for three weeks or a month, we have done the best we can do to prevent stretching of the cicatrix.

**Secretion of Urine after Laparotomy.**—Penrose[2] presents the statistics from 111 cases in which he had opened the abdominal cavity for various causes, giving the amount of urine passed in each case during the first three days after operation. In all of the cases the kidneys seemed to be functionally normal before the operation. A small amount of water was given in case of thirst on the morning of the operation. Small increasing quantities of hot water were given first twenty-four hours after the operation, and food was first administered forty-eight hours after the operation. The average amount of urine passed for all the cases was for first twenty-four hours, 13·4 ozs.; for second twenty-four hours, 14·6 ozs.; and for third twenty-four hours, 19·6 ozs. The patients all recovered easily, never showed any symptoms of ureteral obstruction, or of any renal disturbance.

**Septic General Peritonitis** is, says Oscar Allis,[3] best treated by a thorough cleansing of the peritoneum. Numerous pockets of pus must be opened, and a clean warm fluid carried into every part of the cavity. He can conceive of no adequate provisions for drainage, except a long median incision—kept open by proper packing—with the patient prone except at the time of dressing. To prevent the sides of the incision from closing, or the intestines from uniting to the lips of the incision, a rubber dam thoroughly covered with cerate could be tucked between the abdominal wall and the intestine on each side, with one border emerging from the incision. At the same time to retard, if not prevent, adhesions of the contiguous coils of intestines, the whole serous surface could be well covered with a lubricant like cosmoline or cerate. If the abdomen has been previously flushed with water, the cavity should be dried before the application of the cerate.

**The Value of Bacteriology** in abdominal surgery has been put to a practical use by Penrose,[4] who, whenever possible, has at hand facilities for making immediate bacteriological examinations in all abdominal sections. The object of this examination is to determine the septic or aseptic character of any fluid found in the peritoneal cavity. The result of the examination decides the matter of drainage.

**Embolism and Thrombosis of the Mesenteric Blood vessels.**—Watson[5] reports such a case in a woman, fifty years of age, who had a history of dyspnœa and palpitation extending over several years. One week previous to admission she was seized with violent colicky pains in the abdomen, persisting intermittently for five days, and then becoming localised in the right iliac fossa, but diminished in severity. Vomiting commenced on the fifth day of the illness, and persisted. There had been gradually-increasing abdominal distension from the first, and there had been no passage of flatus or fæces since the commencement of the attack. On opening the abdomen a large amount of dark bloody serum was found free in the peritoneal cavity. The ascending colon was adherent to the parietal peritoneum, and the greater part of it was occupied by hæmorrhagic extravasations beneath its serous surface. Here and there were about a dozen spots of commencing gangrene, and in the mesentery of this portion were felt a large number of apparent thrombi of the superior mesenteric artery or vein. No occlusion was found. An artificial anus was formed, as the patient's condition did not warrant resection, but she died five days later. With regard to diagnosis, the author says there are certain symptoms which, when associated, are fairly, though not positively, characteristic: (1) Colicky, very intense, not definitely localised, abdominal pain; (2) bloody diarrhœa; (3) subnormal temperature. Vomiting, if present (and next to pain it is the most frequent symptom), strengthens the diagnosis, as do also abdominal distension and marked prostration. The author says it may be concluded that laparotomy is indicated in all cases in which the symptoms suggest the nature of the mischief, unless the patient is too greatly prostrated or has some other fatal disease. Resection with immediate suture is recommended where possible. Elliott[6] also believes that immediate suture of the cut ends is the best method of treatment, and quotes two cases, in one of which he resected four feet of intestine, and in the other he made an artificial anus. In conclusion he remarks: As occlusion of the mesenteric vessels is usually associated with heart disease or atheromatous arteries or cirrhosis of the liver, we must not expect much from operative treatment. Nevertheless, patients with symptoms of intestinal obstruction cannot be left unrelieved. Possibly more cases will appear with purely local lesions. They may offer a chance for an occasional surgical succsss.

**Traumatic Intra-abdominal Lesions** are so fatal that Deaver[7] thinks there can hardly be any question as to the wisdom of opening the abdominal cavity. When a diagnosis is impossible, abdominal section is justifiable only when it becomes the last and only chance for the patent. The most prominent symptom in these cases is pain, which is accompanied by shock dependent upon the extent of the injury and the temperament of the individual. Manley,[8] judging from collected reports of laparotomies in cases of abdominal contusions with internal complications, thinks that the cases which demand surgical interference are so commonly depressed from general shock that major operations are generally fatal. All surgeons are agreed that to operate in shock is not permissible; that diagnosis is attended with great difficulties; that after peritonitis, with free extravasation, laparatomy will avail little, even if it does not suddenly cut short life. Chaput[9] thinks that abstention from intervention should be the rule in cases of severe shock unless there are distinct symptoms of hæmorrhage which call for immediate operation; on the other hand, operation is indicated by a previously existing shock (unless the state of the pulse or temperature be unfavourable), by abdominal pain, retraction of the abdomen, nausea, and bilious vomiting. Delorme[10] has had during the past seven years about twenty cases of abdominal contusion, among which were nine of severe contusion of the antero-lateral walls. Of these nine cases only two were subjected to operation; the other seven recovered spontaneously, in spite of their having at first presented very serious symptoms. Michaux[11] argues strongly that it is unquestionable that immediate exploratory laparotomy should be the rule for treatment of contusions of the abdomen, but in order to ensure good results the operation must be performed as early and as rapidly as possible, without neglecting the rules of asepsis, or omitting to make the necessary investigations. He has operated within the first twenty-four hours on six patients, none of whom died; four times the operation was delayed to the twenty-third and twenty-sixth hour, third and seventeenth day respectively, and two of these patients died. Sorge[12] gives notes of fourteen cases of abdominal injury (stabs, gunshot wounds, &c.), in which he performed laparotomy as soon as possible after the injury. Nine out of the fourteen recovered. In four only of the fourteen cases was the laparotomy a simple exploration. The author advises laparotomy in all cases when it is clear that the wound has penetrated the abdomen, unless peritonitis has set in, when operation does more harm than good. In the case of gunshot wounds, if in doubt as to whether internal organs are injured or not, he advises laparotomy. Battle[13] is of opinion that abdominal section should be performed if the shock from which the patient is suffering is considerable and the amount of blood in the peritoneum large, or if the shock, whatever its degree, is increasing in spite of the usual remedies, and continued escape of blood into the peritoneum is taking place. When there is no evidence that the source of blood is a rupture of one of the viscera, it is permissible to make a longer delay. Here also there should be distinct evidence of increasing effusion, and it is not always necessary to search for the bleeding point, although it is desirable that such should be secured if found easily. The author does not think that the question of operation is settled when the hæmorrhage has stopped, though there is generally rapid absorption of the effusion, for a quantity of blood in the peritoneum may cause inflammation from the changes which ensue. One of the first signs which may be called unfavourable will then be a commencing distension of the abdomen, and this should be regarded as an important indication for interference. Chaput[14] and Reclus[15] are in favour of early intervention in cases of abdominal injury, but Berger[16] thinks it would be dangerous to set up laparotomy as a general rule in treating perforating wounds of the abdomen, and that a careful consideration of the existing symptoms and conditions is the sole guide for resorting to, or abstaining from, intervention. Manley[17] records a unique case of rupture of the thoracic duct in a man thirty-five years of age, who was struck in the epigastrium by a heavy pole soon after breakfast. There was great pain and shock, the urine had a slight tinge of blood, but there was no vomiting. On the third day there was marked peritonitis, but about six days later the symptoms began to ameliorate. There was then noticed a distinct bulging forward of the abdominal wall in the right iliac region, which gave a sense of fluctuation on pressure. It was dull on percussion, and an exploring needle showed it to contain a milky chylous fluid. Sixteen ounces of this fluid escaped when the swelling—which was lodged immediately behind the peritoneum—was incised. A drainage tube was inserted, and the man subsequently made a complete recovery.

**Pelvic Hydatids** are rare as compared with abdominal hydatids. Harrison[18] reports the case of a man fortyfive years of age who had never suffered any illness before the present. Nine years ago he began to have trouble about the bladder and rectum. On examination a very large tumour was found occupying the pelvis and lower part of the abdomen, extending almost to the umbilicus. There was great difficulty and pain in urination and defæcation. An incision was made through the perinæum, corresponding to a lateral lithotomy, but without opening the urethra. Half a gallon of fluid and cysts was evacuated, and the cavity was washed out and drained. The patient gradually recovered. The question for consideration in such-like pelvic hydatids is as to where the opening should be made—whether through the abdominal wall, through the rectum, or by incision through the perinæum. Targett[19] supports the view that pelvic hydatid cysts occurred, not in the recto-vesical pouch, but between the muscular coat of the bladder and the sheath of recto-vesical fascia, by three arguments: (1) On careful dissection, the cyst was found to be situated between the muscular wall and the recto-vesical fascia. This fascia intervened between the bladder in front and the vesiculæ seminales and vasa deferentia behind; hence the vasa deferentia and vesiculæ seminales were displaced backwards by these hydatids. (2) Experimentally, the recto-vesical fascia can easily be separated from the muscular coat of the bladder. In an artificial recto-vesical cyst thus produced the vasa deferentia and vesiculæ seminales were displaced backwards just as is the case in hydatids. (3) Sacculi and cystic tumours of the bladder displace the vesiculæ seminales and vasa deferentia in the same way as pelvic hydatids. The author does not think these pelvic hydatids are secondary to hydatids elsewhere; they may be coeval, but not secondary.

[1] Annals of Surgery, April, 1895. [2] Annals of Surgery, Feb.; 1895. [3] Annals of Surgery, Feb., 1895. [4] Therapeutic Gazette, March 15th, 1895. [5] Amer. Journ. Med. Sci., March, 1895, and Boston Med. and Surg. Journ., December 6th, 1894. [6] Annals of Surgery, Jan., 1895, and Amer. Journ. Med. Sci., March, 1895. [7] Annals of Surgery, March, 1895, and N. Y. Med. Journ., March 9th, 1895. [8] Therapeutic Gazette, Jan. 15th, 1895, p. 12. [9] Med. Week, April 5th, 1895. [10] Ibid. [11] Med. Week, March 29th, 1895, and April 12th, 1895. [12] Brit. Med. Journ., May 25th, 1895, and Rif. Med., March 26th, 1895. [13] Lancet, Jan. 26th, 1895, p. 219. [14] Med. Week, Feb. 1st, 1895. [15] Med. Week, Feb. 8th, 1895. [16] Ibid. [17] Annals of Surgery, Feb., 1895. [18] Lancet, April 13th, 1895. [19] Brit. Med. Journ., April 13th, 1895, p. 813.

